# Bacteriological Profile and Outcome of Culture-Positive Neonatal Sepsis in a Special Newborn Care Unit Setting, Odisha

**DOI:** 10.7759/cureus.25539

**Published:** 2022-05-31

**Authors:** Nirmal K Mohakud, Jyoti P Mishra, Manas K Nayak, Jayanti Mishra, Lingaraj Pradhan, Subhra Snigdha Panda, Manas Ranjan Bahera, Rishabh Pugulia

**Affiliations:** 1 Pediatric Medicine, Kalinga Institute of Medical Sciences, Bhubaneswar, IND; 2 Pediatrics, Health & Family Welfare, Bhubaneswar, IND; 3 Pediatric Medicine, Kalinga Institute of Medical Sciences, Bhubaneshwar, IND; 4 Physiology, Kalinga Institute of Medical Sciences, Bhubaneswar, IND; 5 Pediatric Medicine, Capital Hospital, Bhubaneswar, IND; 6 Medical Microbioly, Kalina Institute Of Medical Sciences, Bhubaneswar, IND; 7 Microbiology, Kalinga Institute of Medical Sciences, Bhubaneswar, IND; 8 School of Public Health, KIIT University, Bhubaneshwar, IND; 9 Pediatrics, Kalinga Institute of Medical Sciences, Bhubaneswar, IND

**Keywords:** multi-drug resistant, culture positive, sncu, neonate, sepsis

## Abstract

Introduction: Nearly one-third of neonatal mortality in India is due to neonatal sepsis and death occurs in 30% of culture-positive neonates. Pathogens such as *Klebsiella pneumoniae *and *Escherichia coli *are the most common bacteria responsible for neonatal sepsis in India and South Asia.

Materials and Methods: It was an observational study, conducted in special newborn care units (SNCUs) of Capital Hospital in Bhubaneswar, Odisha from May 2017 to October 2019. All neonates (<28 days of life) with blood culture-positive sepsis were included in this study. Blood cultures were sent in all the babies with features of clinical sepsis. The demographic profile of neonates, clinical presentations, isolated organisms, and their sensitive patterns was recorded for analysis.

Results: Blood culture was sent in 445 suspected neonates with clinical sepsis out of which 115 blood culture positive organisms were isolated. Among the isolated organisms, 42 (35.6%) cases were *Staphylococcus aureus* followed by* Coagulase negative Staphylococcus *(CONS) (20.8%), *E. coli *(19.1%), *K. pneumoniae* (10.4%), *Acinetobacter baumannii *(2.7%), *Enterobacter *spp.(4.3%), *Enterococcus *spp. (4.3%), and *Pseudomonas aeruginosa *(2.7%). *S. aureus* was the predominant organism found in both early and late-onset sepsis. All Gram-negative bacilli (GNB) are resistant to ampicillin whereas cephalosporin resistance was found in 68% of cases. Mortality due to sepsis was 8%.

Conclusion: *S. aureus *followed by CONS was found to be the most common cause of sepsis in SNCU. A high degree of resistance of organisms to penicillins and cephalosporins calls for a re-evaluation of antibiotic policy and protocols for empirical treatment in neonatal sepsis.

## Introduction

Nearly one-third of neonatal mortality in India is due to neonatal sepsis and death occurs in 30% of culture-positive neonates [1-2]. Neonatal sepsis is classified as early onset sepsis (EOS) (<72 h) and late onset sepsis (LOS) (>72 h) based on the onset of illness. EOS occurs usually due to pathogens present in the genital tract of the mother whereas LOS occurs due to pathogens acquired either from the hospital or from the community. There is a gradually increasing trend of multi-drug resistant (MDR) pathogens in tertiary care neonatal intensive care units (NICU) and special newborn care units (SNCUs) all over India. Multi-drug resistance was defined as the acquired resistance to at least one agent in three or more antimicrobial categories as per the Centers for Disease Control and Prevention (CDC) guidelines [3]. Strict antibiotics stewardship program will enable us to counteract multi-drug resistance patterns of emerging pathogens. The major hallmark of antibiotic stewardship is to identify the isolated culture-positive organisms and their antibiotic sensitivity pattern. The prevalence of organism in SNCUs also differ from tertiary care NICUs in our country and it is also different from that of the Western world. Strict monitoring of bacterial flora and the resistance pattern of a unit are always required as both change very frequently. Pathogens such as *Klebsiella pneumoniae* and *Escherichia coli* are the most common cause of neonatal sepsis in India and South Asia [4-5]. The majority of studies were done in tertiary care units with SNCU hardly contributing to it. Gradually increasing trends of MDR strains in our SNCU prompted us to do this study to evaluate the isolated organism and their antibiogram pattern in neonatal sepsis.

## Materials and methods

This was a hospital-based observational study conducted by Capital Hospital and Kalinga Institute of Medical Science, Bhubaneswar, Odisha from May 2017 to October 2019. All neonates (<28 days of life) with blood culture positive sepsis in SNCU of Capital Hospital, Bhubaneswar were included in this study after institutional ethics committee approval (KIIMS/KIIT/IEC/83/2017). Neonates with congenital malformations were excluded from the study. Blood cultures were sent from all the babies of SNCU with signs and symptoms of sepsis-like lethargy, refusal feeding, breathing difficulty, poor perfusion, seizures, and temperature instability or in any baby admitted with maternal risk factors like foul-smelling liquor, chorioamnionitis, and prolonged rupture of membrane for >24 h [6]. Two milliliters of blood were collected from peripheral blood with all aseptic measures in BACT/ALERT PF plus a pediatric blood culture bottle. Blood culture was done by an automated method in BACT/ALERT 3D culture system (bioMerieux, Durham, NC, USA). Diagnosis of culture-positive sepsis was confirmed after isolation of microorganisms in suspected cases of clinical sepsis [5, 7]. Identification and antibiotic sensitivity of isolated bacteria were done by VITEK 2 Compact Automated ID/AST instrument (bioMerieux) and interpretation was done as per the guidelines of the Clinical & Laboratory Standards Institute guidelines [8]. Dehydrated media and antibiotic disks were procured from Hi Media Laboratories Pvt Ltd (Mumbai, India). Disk diffusion method using ceftazidime (30 μg), cefotaxime (30 μg), ceftazidime plus clavulanic acid (30/10 μg), and cefotaxime plus clavulanic acid (30/10 μg) combination was done for the detection of extended spectrum beta lactamase (ESBL) producing strains [8]. Strains of *S. aureus* resistant to the majority group of antibiotics such as β-lactams (penicillins, cephalosporins, and carbapenems) were defined as methicillin resistant *Staphylococcus aureus* (MRSA) [9].

Statistical analysis

Demographic profiles such as gestation, sex, birth weight, day of onset of illness, inborn/outborn cases, isolated organisms, and their antibiogram pattern were recorded for analysis using Microsoft Excel. Multi-drug resistance pattern among each Gram-negative and Gram-positive species was described.

For data collection, Microsoft Excel 2013 (Microsoft, Redmond, WA, USA) was used whereas descriptive statistics like percentages and means ± standard deviations were used to describe variables. The SPSS Statistics for Windows, version 21.0 (IBM Corporation, Armonk, NY, USA) was used for statistical analyses.

## Results

Blood culture was sent for 445 suspected neonates with clinical sepsis out of which 115 neonates culture-positive organisms were isolated. Gram-positive organisms were isolated in 60.8% of cases of clinical sepsis. The mean birth weight was 1.84 ± 0.87 kg, and the mean gestational age was 33.47 ± 4.79 weeks in neonates with culture-positive sepsis. Table 1 showed the demographic profile of the culture-positive neonates. 

**Table 1:** Demographic profile of neonates with clinical sepsis (n=445).

Variables	Culture positive sepsis (n=115)	Culture negative sepsis (n=330)	Total (n=445)
Gestation <28 weeks	8 (7%)	20 (6.1%)	28 (6.3%)
28-32 weeks	24 (20.9%)	56 (16.9%)	80 (18%)
32-36 weeks	35 (30.4%)	104 (31.5%)	139 (31.2%)
>37 weeks	48 (41.7%)	150 (44.4%)	198 (44.5%)
Birth Weight <1 kg	11 (9.6%)	26 (7.9%)	37 (8.3%)
1-1.5 kg	31 (26.9%)	98 (29.7%)	129 (29%)
1.5 -2.5 kg	38 (33%)	76(23%)	114 (25.6%)
>2.5 kg	35 (30.5%)	130 (39.4%)	165 (37.1%)
Sex Male	61 (53%)	189 (57.3%)	250 (56.1%)
Female	45 (47%)	141( 42.8%)	195 (43.9%)
Inborn	62 (53.9%)	196 (59.4%)	258 (58%)
Outborn	53 (46.1%)	134 (40.6%)	187 (42%)
Early onset sepsis	32 (27.8%)	122 (37%)	154 (34.6%)
Late onset sepsis	83 (72.2%)	208 (63%)	291 (65.4%)

**Figure 1:**
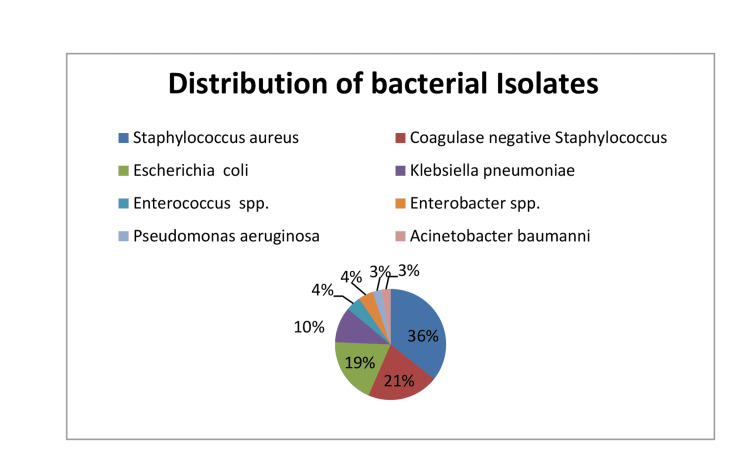
Type and frequency of culture-positive organism: (n=115).

Among isolated organisms, 42 (35.6%) cases were S. aureus followed by *Coagulase negative Staphylococcus *(CONS) (20.8%), *E. coli* (19.1%), *K. pneumoniae* (10.4%), *Acinetobacter baumannii* (2.7%), *Enterobacter* spp. (4.3%), *Enterococcus* spp. (4.3%), and *Pseudomonas aeruginosa* (2.7%). LOS was found to be in 72.2% of cases. *S. aureus* was the most common organism isolated in both EOS and LOS (Figure 1).

Culture-positive neonates most commonly presented with feeding intolerance in 33.9% of cases followed by seizure, shock, pneumonia requiring respiratory support, disseminated intravascular coagulopathy, and acute kidney injury. The majority (75%) of death was due to infections caused by Gram-negative organisms and its complication. Ninety-eight (85.2%) neonates recovered from sepsis and nine babies left against medical advice before completion of treatment.

**Figure 2:**
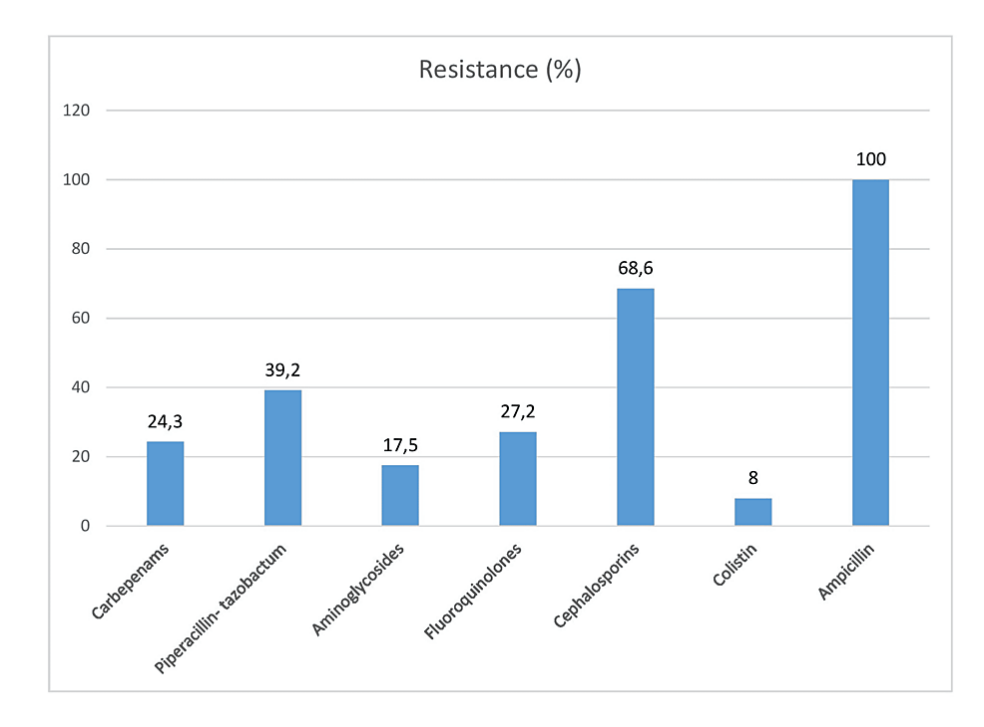
Resistance pattern of Gram-negative organism among culture-positive neonates (n=45).

All Gram-negative organisms were resistant to ampicillin whereas cephalosporin resistance was found in 68% of cases. Out of carbapenem-resistant Gram-negative bacilli, 80% of organisms were sensitive to colistin. ESBL producing bacteria was isolated in 40% of neonates whereas MDR strains were found in 16 (35.2%) cases (Figure 2).

**Figure 3:**
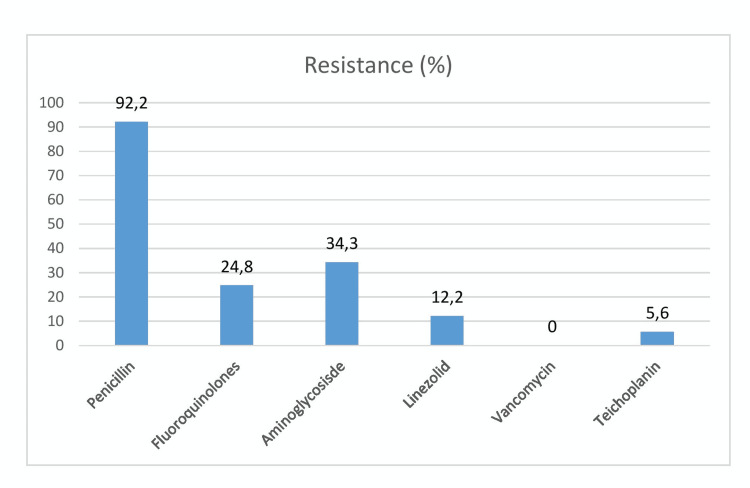
Resistance pattern of Gram-positive organism among culture-positive neonates (n=70).

All Gram-positive organisms are sensitive to vancomycin whereas resistance to penicillin was seen in 92.3% cases. The MRSA-producing strains were isolated in 13 (18%) neonates (Figure 3).

## Discussion

In our study, clinical sepsis was confirmed in 25% of cases with isolation of organisms in blood culture which is similar to other studies [10-12]. As the majority of referral centers do not have facilities for blood cultures, the start of empirical treatment before taking a blood culture in more than 50% of cases may be the reason for sterile culture in most of the cases.

In more than 80% of cases of clinical sepsis, neonates presented with LOS which is consistent with other studies [13-14]. As the majority (60%) of our cohort population were preterms, prolonged hospital stay and use of central catheters frequently for total parenteral nutrition may have led to LOS more often. In our study, more than 60% of neonates received total parenteral nutrition through central venous catheters. Preterm and low birth weight babies were more commonly affected in our study like other studies [14-15]. Preterm low birth weight babies may be 3-10 times more prone to developing sepsis than term neonates [16].

Gram-positive organisms were found in the majority (60%) of the cases similar to findings of other studies [14, 17-19]. But Gram-negative organisms are most commonly found in the majority of studies done in tertiary care centers in India [20-22]. As per NNPD data, *K. pneumoniae* was the most common pathogen (32.5%) of neonatal sepsis, followed by *S. aureus* (13.6%) and *E. coli* (10.6%) [23]. Newly emerging pathogens of tertiary care NICUs like non-fermenting Gram-negative bacilli were found to be very few in our study unlike other studies [24]. The main reason for it may be due to lack of mechanical ventilation and other interventions as it is an SNCU with a level II facility. In our study, *S. aureus* was found to be the most common Gram-positive organism followed by CONS, but MRSA strain was found to be an important pathogen associated with endemic infections in other NICU population [17, 19, 25]. Isolation of CONS in 20% of cases in our study may be due to contamination during sampling or during birth from the mother’s birth canal. Hence healthcare staff education and infection control guidelines should be reinforced. S. aureus was isolated in the majority of cases in both EOS and LOS out of which MRSA was isolated in 18% of cases similar to other studies [14, 26]. It could be due to poor personal hygiene. Isolation of S. aureus in inborn cases may be the result of a lack of strict hand washing, infrequent or improper sterilization, and cleaning of ICU.

Gastrointestinal symptoms like feeding intolerance were the most common modes of presentation in both EOS and LOS in our study whereas in other studies respiratory distress, lethargy, and poor cry were more common [24, 27-28]. Due to the predominant incidence of LOS in our study, respiratory distress may be a less common mode of presentation which is seen frequently with EOS.

In our study, Gram-negative and Gram-positive organisms were resistant to ampicillin in 100% and 92% cases, respectively. Resistance of Gram-negative organisms to cephalosporins was found in 70% of cases which is similar to the resistance pattern found in a study by Pavan Kumar et al. [19]. Starting of antistaphylococcal antibiotics as empirical therapy should be considered in view of the high prevalence of *Staphylococcus* as a causative organism in both EOS and LOS. Due to gradually increasing resistance to extended-spectrum cephalosporins, there is a need to change the choice of empirical antibiotics policy.

The MDR strains were found in 35% of cases which is lower than MDR strains found in other studies [5, 21]. The prevalence of ESBL producing bacteria in our study was 40% which is lower than other studies in India [29-30]. Mortality from culture-positive sepsis in our study was 7% which is lower than in other studies done in tertiary care centers in India [5, 22, 24]. A possible explanation for lower MDR strains with ESBL producing bacteria and low mortality may be that few critical cases are left against medical advice or referred to other hospitals due to the requirement of ventilator support and as it is a level II NICU, very sick outborn neonates were not admitted.

The limitations include as it is a level II NICU, many critically ill neonates requiring ventilator support were referred to other tertiary care NICUs or left against medical advice. Hence our estimate of the morbidity pattern and mortality rate may have been low.

## Conclusions

It is important to analyze the blood culture report and its sensitivity pattern as well as to formulate local antibiotic usage for better clinical outcomes. *S. aureus* followed by CONS were found to be the most common cause of sepsis in SNCUs. A high degree of resistance of organisms to penicillins and cephalosporins calls for a re-evaluation of antibiotic policy and protocols for empirical treatment in neonatal sepsis. Infection control guidelines and education of healthcare staff about frequent hand washing should be reinforced. Strict antibiotic stewardship should be practiced to save the babies from the development of multi-drug resistance in the future.
